# Supplementary material for: Integrative epigenomic and transcriptomic analysis reveals the requirement of JUNB for hematopoietic fate induction
Source: Nat Commun. 2022 Jun 6;13:3131. doi: 10.1038/s41467-022-30789-4 (PMC9170695; doi:10.1038/s41467-022-30789-4)
Supplement: Supplementary file 1 — Supplementary Information [file 41467_2022_30789_MOESM1_ESM.pdf]

Supplementary Information

Integrative epigenomic and transcriptomic analysis reveals the requirement of JUNB for hematopoietic fate induction

Xia Chen, Peiliang Wang, Hui Qiu, Yonglin Zhu, Xingwu Zhang, Yaxuan Zhang, Fuyu Duan, Shuangyuan Ding, Jianying Guo, Yue Huang and Jie Na

Supplementary Figure 1

Supplementary Figure 2

Supplementary Figure 3

Supplementary Figure 4

Supplementary Figure 5

Supplementary Figure 6

Supplementary Figure 7

Supplementary Table 1

Supplementary Table 2

Supplementary Table 3

Supplementary Table 4

Supplementary Fig. 1

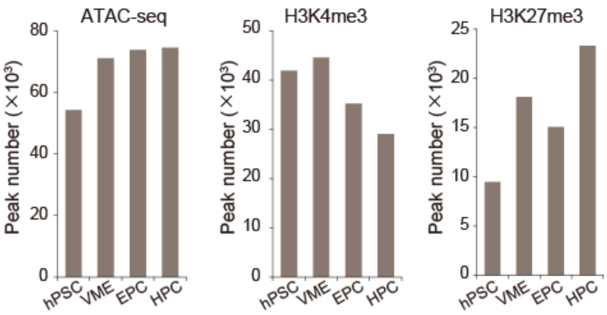

Supplementary Fig. 1. Dynamic change of chromatin status during HPC differentiation.

Number of peaks identified in ATAC-seq, H3K4me3 and H3K27me3 ChIP-seq, respectively. hPSC, human pluripotent stem cell. VME, vascular mesoderm cell. EPC, endothelial progenitor cell. HPC, hematopoietic progenitor cell. Source data are provided as a Source Data file.

Supplementary Fig. 2

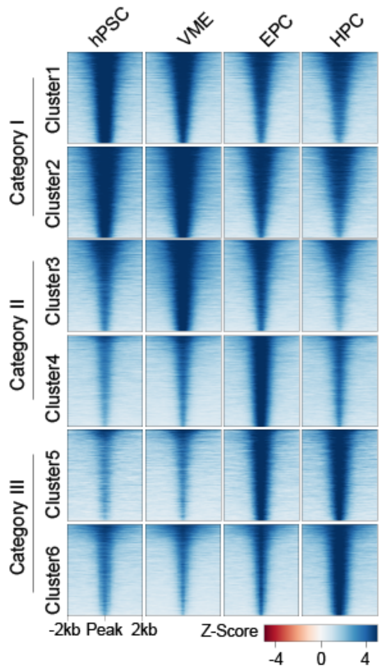

Supplementary Fig. 2. Temporal chromatin accessibilities during HPC differentiation.

Heatmap showing ATAC-seq peaks in hPSC, VME, EPC and HPC clustered by Mfuzz. hPSC, human pluripotent stem cell. VME, vascular mesoderm cell. EPC, endothelial progenitor cell. HPC, hematopoietic progenitor cell.

Supplementary Fig. 3

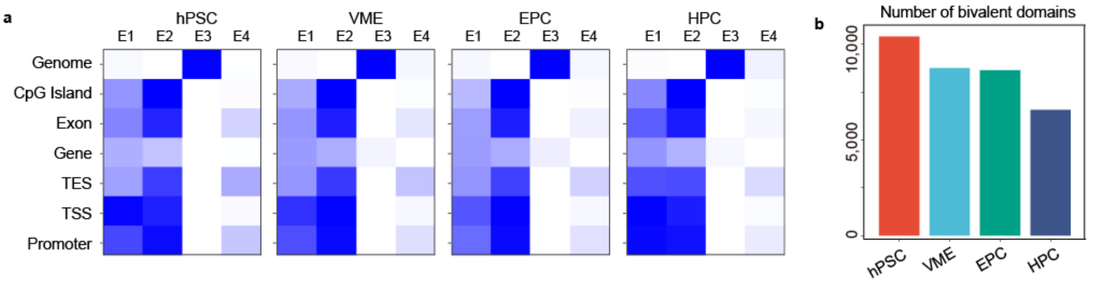

c

Table:Dynamic change of bivalent domains

Stage transition	turn to be H3K4me3-only	stable bivalent	turn to be H3K27me3-only	turn to be unmarked
hPSC ⟶ VME	21%	55%	22%	2%
VME ⟶ EPC	19%	64.3%	16%	0.7%
EPC ⟶ HPC	7%	59%	33%	1%

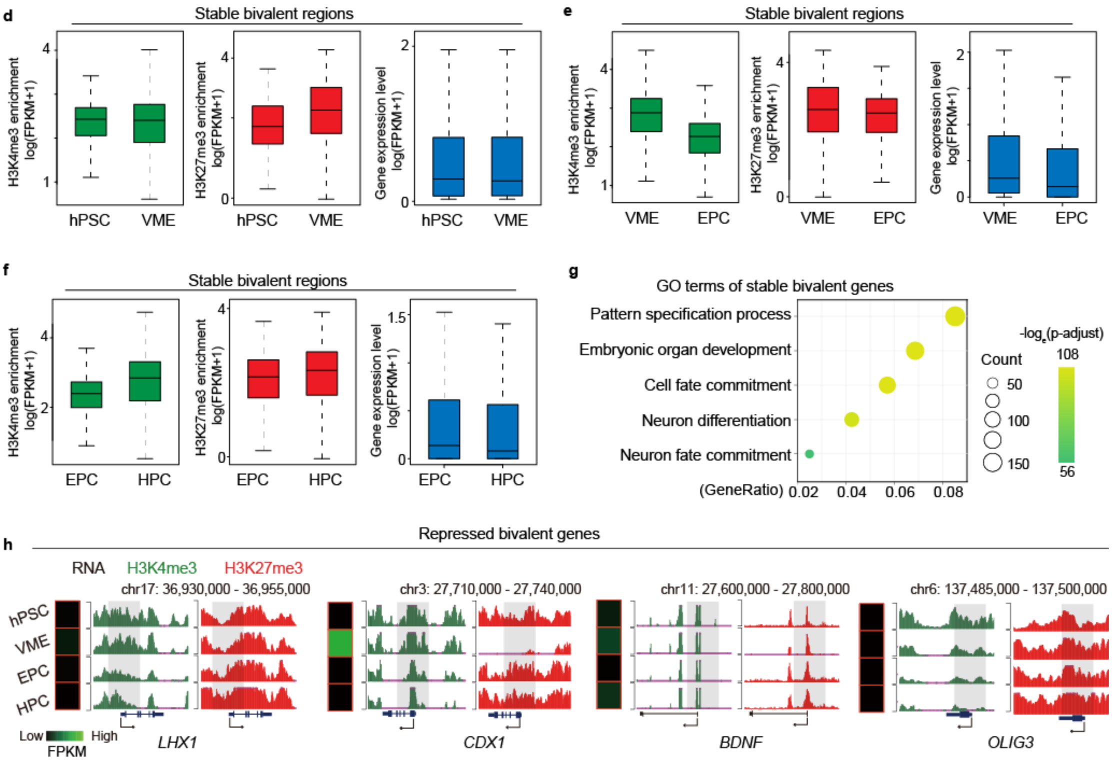

Supplementary Fig. 3. Dynamics of stable bivalent domains during hPSC-HPC differentiation.

a. Genomic annotation of four chromatin states in each stage during differentiation, each column corresponds to one of chromatin states identified in Fig.3a. b. The number of bivalent domains in hPSC, VME, EPC, and HPC. c. The percentage of bivalent domain change between successive cell states. d-f. The H3K4me3 (left), H3K27me3 (middle) signal intensity and their associated gene expression(right) of stable bivalent marked regions during the hPSC differentiation into VME (d, n = 2,655), VME to EPC

(e, n = 2,607) and EPC to HPC (f, n = 2,573), and the signal density was calculated as H3K4me3 and H3K27me3 ChIP-seq RPKM. g. GO terms of stable bivalent domain associated genes from EPC to HPC. *p* values, Fisher's Exact test. h. UCSC genome browser snapshots showing H3K4me3 and H3K27me3 modification profiles of the selected genes. The putative promoters are shaded. The normalized RNA-seq FPKM for each gene at different time points are shown on the left. The view scale of the genome browser is adjusted according to the global data range. hPSC, human pluripotent stem cell. VME, vascular mesoderm cell. EPC, endothelial progenitor cell. HPC, hematopoietic progenitor cell. Source data are provided as a Source Data file.

Supplementary Fig. 4

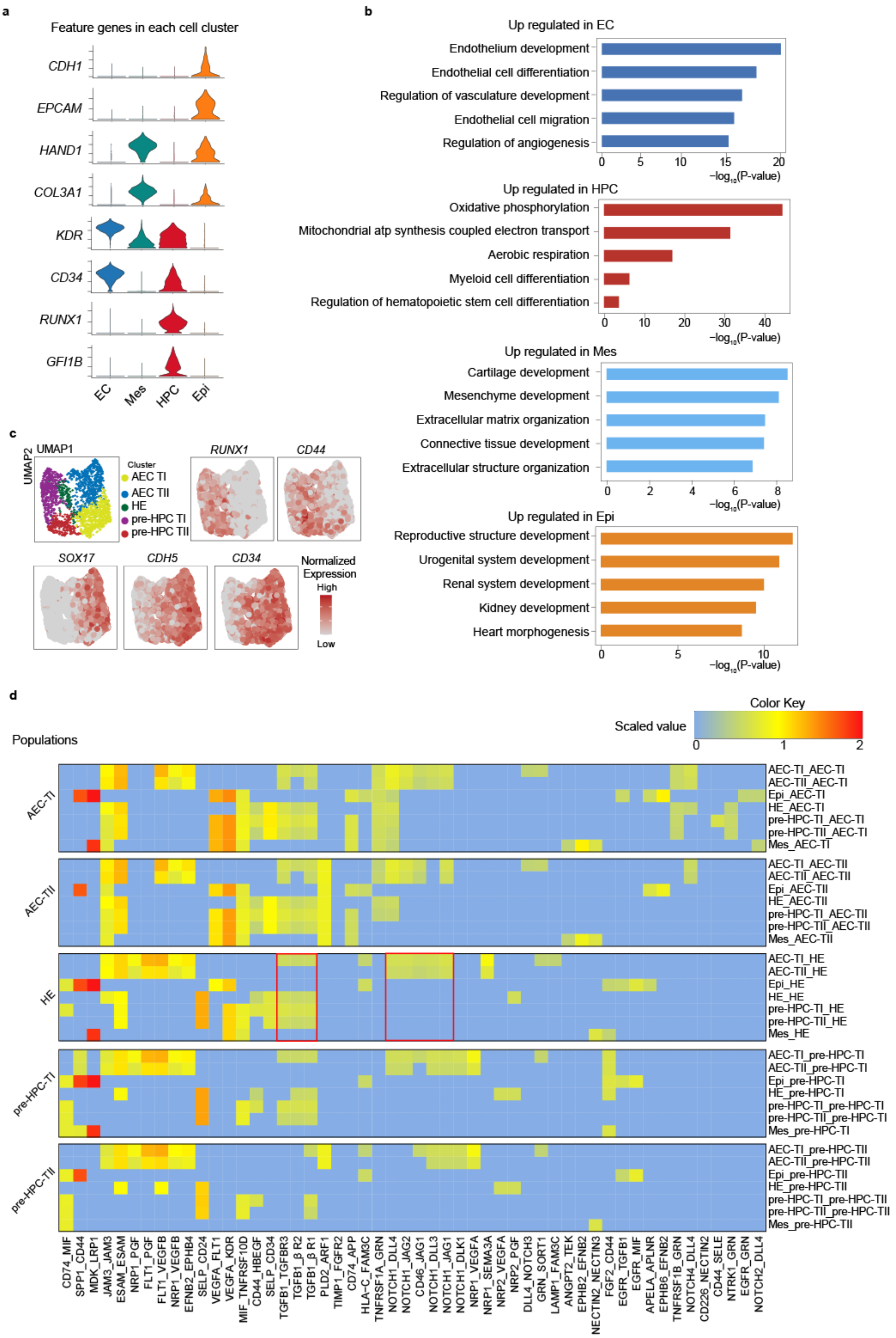

Supplementary Fig. 4. ScRNA-seq and scATAC-seq analysis revealed subpopulations of ECs and early HPCs.

a. The expression profiles of selected key genes representing each cell cluster. b. Statistically over-represented GO and biological process terms enriched in the DEGs of each cell type. *p* values, Fisher's Exact test. c. The scatter plots showing the expression and distribution of SOX17, CDH5, RUNX1, CD44 and CD34. d. Ligand-receptor analysis of Epi, Mes, AEC TI, AEC TII, pre-HPC TI, pre-HPC TII, and HE clusters. Epi, epithelial cell. Mes, mesoderm cell. EC, endothelial cell. HPC, hematopoietic progenitor cell. HE, hemogenic endothelium. AEC, arterial endothelial cell. AEC TI, AEC-type I. AEC TII, AEC-type II. pre-HPC TI, pre-HPC type I. pre-HPC TII, pre-HPC type II. Source data are provided as a Source Data file.

Supplementary Fig. 5

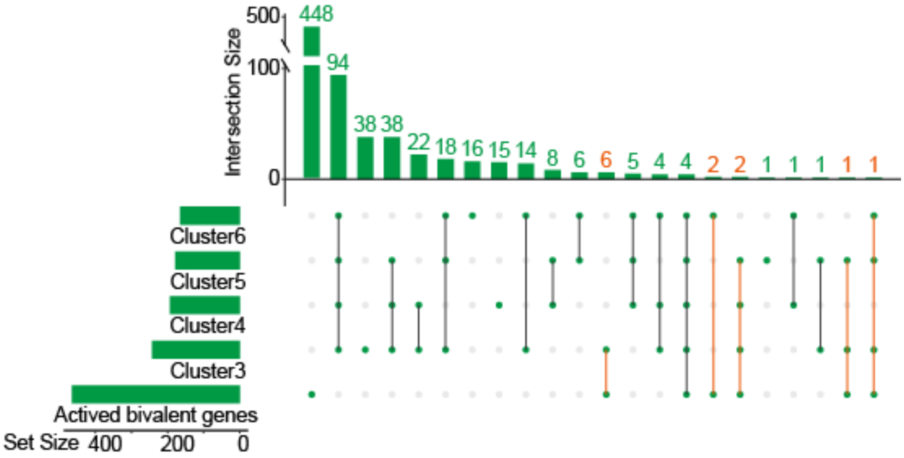

Supplementary Fig. 5. Epigenetic profiling identified potential regulators driving hematopoietic specification.

UpSetR diagram visualizing intersections between TF motifs enriched in category II and category III ATAC-peaks as indicated in Fig. 2g and activated bivalent genes. The red lines indicate TFs with binding motifs enriched in corresponding categories, which are also activated bivalent genes. Source data are provided as a Source Data file.

Supplementary Fig. 6

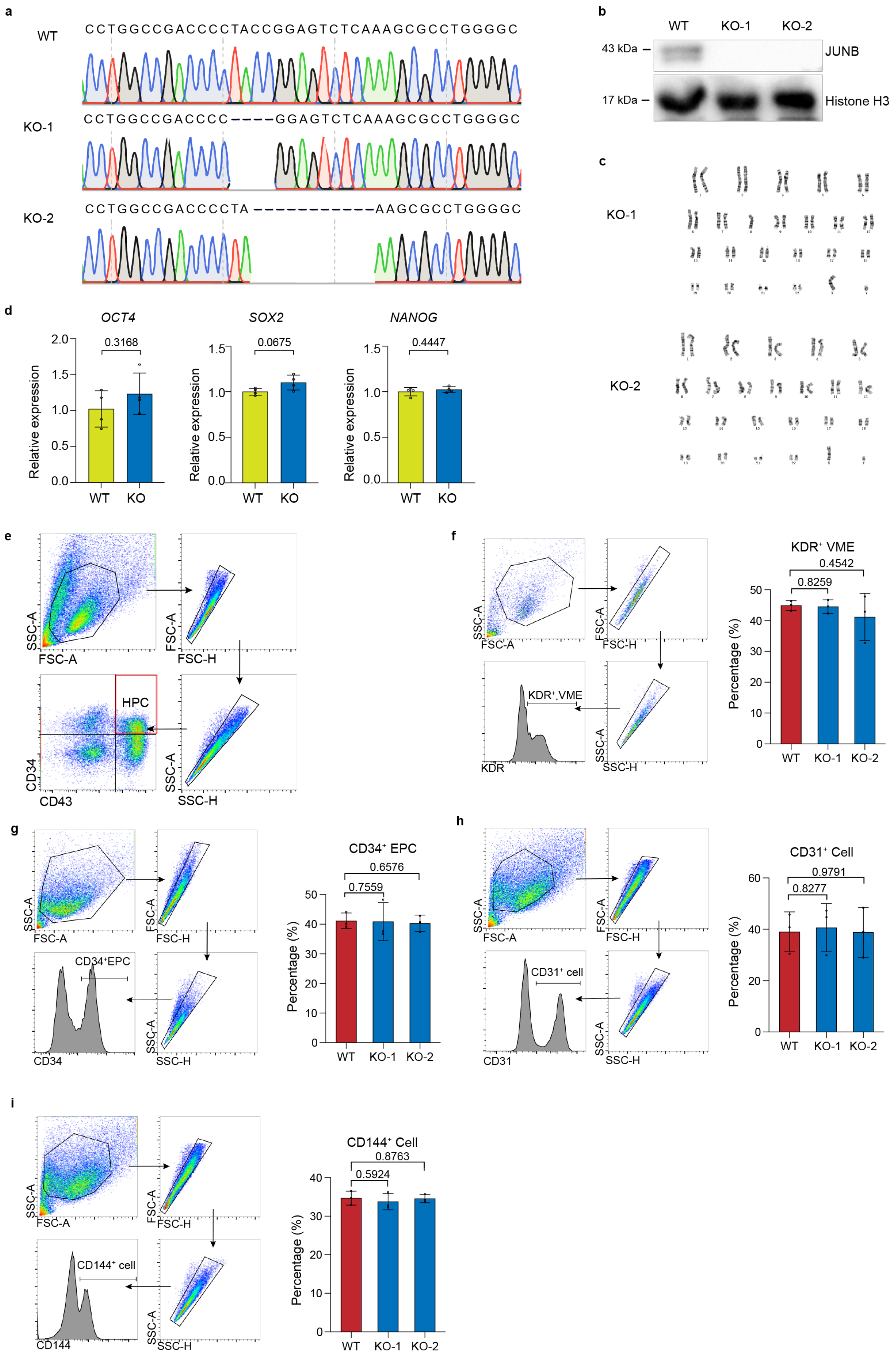

Supplementary Fig. 6. JUNB deficiency severely impaired the generation of HECs and HPCs.

a, b. *JUNB* KO are verified by Sanger sequencing (a) and western blot (b). Images of immunoblotting are representative of three independent experiments. c. Normal karyotype of *JUNB* KO cell lines used in this study. d. Expression of *OCT4*, *NANOG*, and *SOX2* in WT and *JUNB* KO cell lines. n = 3 independent experiments. Data are presented as mean values ± SD. *p* values, two-tailed unpaired Student's t test. e, Flow cytometric sorting strategy for the characterization of HPC population. f-i. Flow cytometric gating scheme and quantification of VME (KDR^+) population on day3 (f, n = 3 independent experiments), $CD34^+$ population (g, n = 3 independent experiments), $CD31^+$ population (h, n = 3 independent experiments) and $CD144^+$ population (i, n = 3 independent experiments) on day6 in WT and *JUNB* KO cells in WT and *JUNB* KO group, respectively. Data are presented as mean values ± SD. *p* values, two-tailed unpaired Student's t test. VME, vascular mesoderm cell. EPC, endothelial progenitor cell. Source data are provided as a Source Data file.

Supplementary Fig. 7

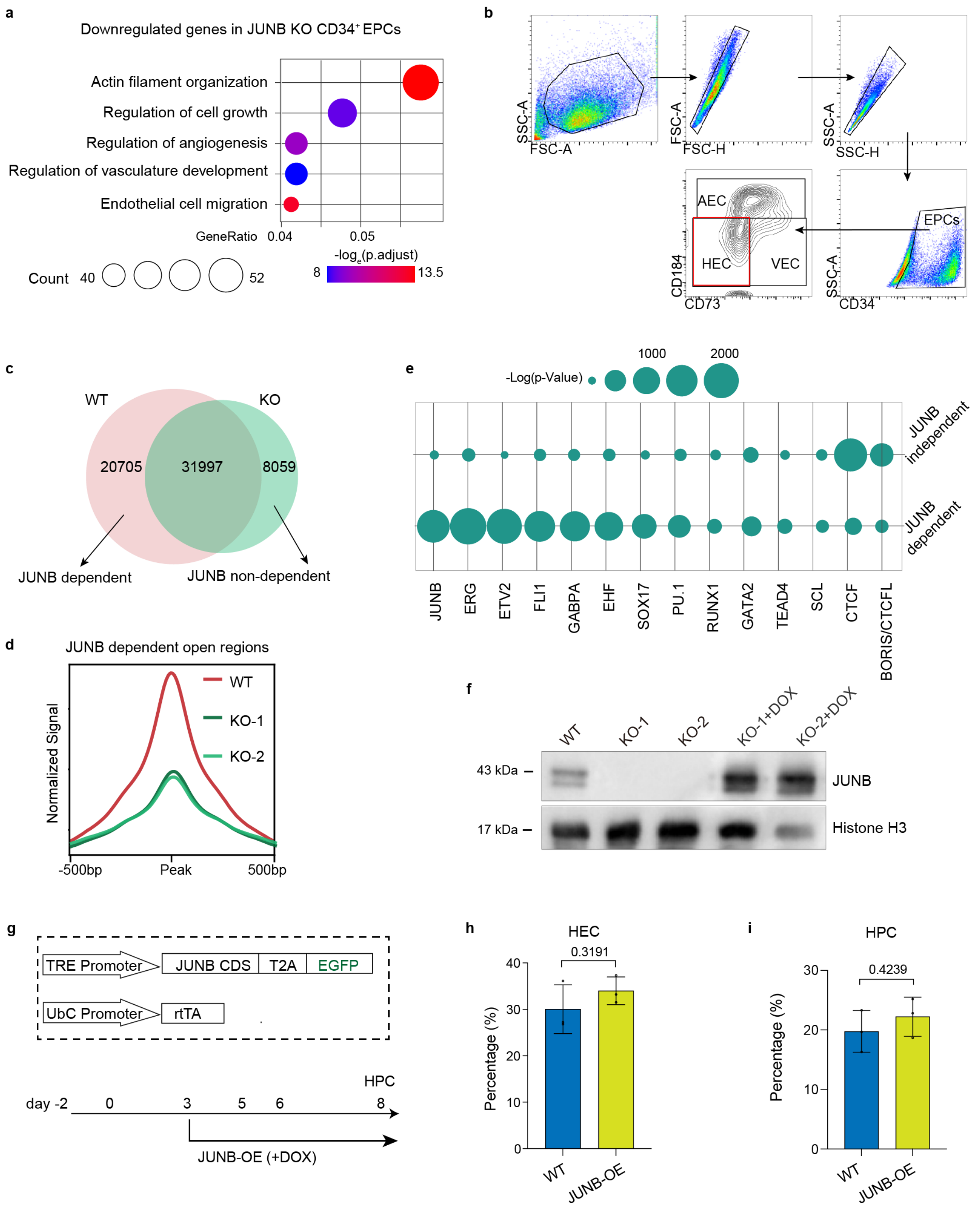

Supplementary Fig. 7. JUNB functions in HEC and HPC specification.

a. Top GO terms of downregulated genes in *JUNB* KO CD34^{+} EPCs. The dot color shows the enrichment from Fisher's Exact *p* value. b. Flow cytometric sorting strategy for the characterization of HEC population. c. Venn plot showing number of ATAC-seq peaks in WT and *JUNB* KO HECs, respectively. d. Metaplot showing the levels of ATAC-seq signals at JUNB dependent sites in WT and *JUNB* KO HECs. e. Plot

showing TF motif enrichment from JUNB dependent peaks. The dot size shows the enrichment from Fisher's Exact *p* value. f. Immunoblotting for JUNB in *JUNB* KO cell lines and rescue cell lines. Images are representative of three independent experiments. g. Schematic illustrating the *JUNB* inducible expression constructs and the induction time window. h, i. Bar plots showing the percentage of HEC ($CD34^+$ $CD73^-$ $CD184^-$) (n = 3 independent experiments) and HPC ($CD34^+$ $CD43^+$) (n = 3 independent experiments) on differentiation day 6 (h) and day 8 (i) in WT and *JUNB* OE cells. Data are presented as mean values ± SD. *p* values, two-tailed unpaired Student's t test. EPC, endothelial progenitor cell. HEC, hemogenic endothelial cell. HPC, hematopoietic progenitor cell. Source data are provided as a Source Data file.

Supplementary Table 1. Antibodies used in this study.

Antibodies	Cat. No.	Company	Origin	Ig type	Application	Dilution
Anti-CD31-FITC	555445	BD	Mouse	mAb, IgG	FACS	1:100
Anti-CD34-APC	555824	BD	Mouse	mAb, IgG	FACS	1:100
Anti-CD43-FTIC	315203	BioLegend	Mouse	mAb, IgG	FACS	1:100
Anti-CD43-PE	343204	BioLegend	Mouse	mAb, IgG	FACS	1:100
Anti-CD73-PE	344003	BioLegend	Mouse	mAb, IgG	FACS	1:100
Anti-CD184-APC/Cy7	306527	BioLegend	Mouse	mAb, IgG	FACS	1:100
Isotype Control Antibody-APC	130-113-831	Miltenyi Biotec	Mouse	mAb, IgG	FACS	1:100
sotype Control Antibody-PE	130-113-834	Miltenyi Biotec	Mouse	mAb, IgG	FACS	1:100
Anti-JunB	ab128878	Abcam	Rabbit	mAb, IgG	western blot	1:1,000
Anti-Histone H3	A2348	abclonal	Rabbit	pAb, IgG	western blot	1:10,000
Anti-Rabbit IgG	111-035-144	Jackson ImmunoResearch	Goat	pAb, IgG	western blot	1:2000
Anti-JunB	3753S	Cell Signaling Technology	Rabbit	mAb, IgG	IP	1:50
Anti-H3K4me3	9727	Cell Signaling Technology	Rabbit	pAb, IgG	ChIP	1:50
Anti-H3K27me3	pAb-069-050	Diagnode	Rabbit	pAb, IgG	ChIP	1:50

Supplementary Table 2. DNA oligonucleotide sequences for guide RNA.

Target	Forward	Reverse
gRNA targeting JUNB	GGGTAAAAGTACTGTCCCGG	CCGGGACAGTACTTTTACCC
Non targeting control (NTC)	CACCGATTGTTCGACCGTCTACGGG	AAACCCCGTAGACGGTCGAACAATC

Supplementary Table 3. Primers for genotyping of *JUNB* knockout cell lines.

PCR	Forward	Reverse
JUNB	AGCTACTTTTCTGGTCAGGGCT	TGTCACGTGGTTCATCTTGTG

Supplementary Table 4. Primers for genotyping of *JUNB* knockout cell lines.

Gene	Forward	Reverse
GAPDH	TGATGACATCAAGAAGGTGGTGAAG	TCCTTGGAGGCCATGTGGGCCAT
OCT4	CGACCATCTGCCGCTTTGAG	CCCCCTGTCCCCCATTCCTA
SOX2	GCTACAGCATGATGCAGGACCA	TCTGCGAGCTGGTCATGGAGTT
NANOG	GGATGGTCTCGATCTCCTGA	CCTCCCAATCCCAAACAATA
