# Supplementary material for: Integrative epigenomic and transcriptomic analysis reveals the requirement of JUNB for hematopoietic fate induction
Source: Nat Commun. 2022 Jun 6;13:3131. doi: 10.1038/s41467-022-30789-4 (PMC9170695; doi:10.1038/s41467-022-30789-4)
Supplement: Supplementary file 3 — Description of Additional Supplementary Files [file 41467_2022_30789_MOESM3_ESM.pdf]

Description of Additional Supplementary Files

File Name: Supplementary Data 1
Description: **Gene Ontology (GO) enrichment results.** The tables show Gene Ontology (GO) enrichment results of ATAC-seq peak closest genes (Figure 2d-f), VME or EPC activated bivalent genes (Figure 3e, h), stable bivalent genes (supplementary Figure 3g), and feature genes in each lineage defined by scRNA-seq (supplementary Figure 4b).
