# Supplementary material for: Integrative epigenomic and transcriptomic analysis reveals the requirement of JUNB for hematopoietic fate induction
Source: Nat Commun. 2022 Jun 6;13:3131. doi: 10.1038/s41467-022-30789-4 (PMC9170695; doi:10.1038/s41467-022-30789-4)
Supplement: Supplementary file 5 — Reporting Summary [file 41467_2022_30789_MOESM5_ESM.pdf]

nature portfolio

Corresponding author(s): Jie Na

Last updated by author(s): May 8, 2022

Reporting Summary

Nature Portfolio wishes to improve the reproducibility of the work that we publish. This form provides structure for consistency and transparency in reporting. For further information on Nature Portfolio policies, see our Editorial Policies and the Editorial Policy Checklist.

Statistics

For all statistical analyses, confirm that the following items are present in the figure legend, table legend, main text, or Methods section.

n/a	Confirmed	
☐	☒	The exact sample size (*n*) for each experimental group/condition, given as a discrete number and unit of measurement
☐	☒	A statement on whether measurements were taken from distinct samples or whether the same sample was measured repeatedly
☐	☒	The statistical test(s) used AND whether they are one- or two-sided<br>*Only common tests should be described solely by name; describe more complex techniques in the Methods section.*
☐	☒	A description of all covariates tested
☐	☒	A description of any assumptions or corrections, such as tests of normality and adjustment for multiple comparisons
☐	☒	A full description of the statistical parameters including central tendency (e.g. means) or other basic estimates (e.g. regression coefficient) AND variation (e.g. standard deviation) or associated estimates of uncertainty (e.g. confidence intervals)
☐	☒	For null hypothesis testing, the test statistic (e.g. *F*, *t*, *r*) with confidence intervals, effect sizes, degrees of freedom and *P* value noted<br>*Give P values as exact values whenever suitable.*
☒	☐	For Bayesian analysis, information on the choice of priors and Markov chain Monte Carlo settings
☒	☐	For hierarchical and complex designs, identification of the appropriate level for tests and full reporting of outcomes
☐	☒	Estimates of effect sizes (e.g. Cohen's *d*, Pearson's *r*), indicating how they were calculated

Our web collection on statistics for biologists contains articles on many of the points above.

Software and code

Policy information about availability of computer code

Data collection	No custom software was used in this study.<br>Public datasets were retrieved in this study as described and cited in the main text section.<br>Sequencing was performed using Illumina Hi-seq X Ten and NovaSeq 6000.
Data analysis	For most statistical comparisons, Prism 9 [version 9.2.0 (283)) for macOS was used.<br>FACS were analyzed with FlowJo software (version 10.4).<br>Sequencing data were analyzed as described in the Methods section. Softwares were also include below:<br>Trim Galore v0.6.7<br>bowtie2, v2.3.5<br>MACS2, v2.2.6<br>Homer, v4.10.4<br>deeptools, v3.3.0<br>Mfuzz, v 2.56.0<br>ChromHMM, v1.22<br>samtools, v1.9<br>Picard MarkDuplicates, v2.26.10<br>STAR, v2.7.1a<br>htseq-count, v0.11.1<br>bedtools, v2.27.1<br>DESeq2, v1.32.0<br>clusterProfiler, v3.6.0<br>FastQC, v0.11.9<br>cellranger, v2.0.1

Souporcell, v2.0
Seurat, v3.2

For manuscripts utilizing custom algorithms or software that are central to the research but not yet described in published literature, software must be made available to editors and reviewers. We strongly encourage code deposition in a community repository (e.g. GitHub). See the Nature Portfolio guidelines for submitting code & software for further information.

Data

Policy information about availability of data

All manuscripts must include a data availability statement. This statement should provide the following information, where applicable:
- Accession codes, unique identifiers, or web links for publicly available datasets
- A description of any restrictions on data availability
- For clinical datasets or third party data, please ensure that the statement adheres to our policy

The high-throughput sequencing data and single cell data generated in this study have been deposited in the GEO repository database under accession number GSE168372 (https://www.ncbi.nlm.nih.gov/geo/query/acc.cgi?acc=GSE168372).

Field-specific reporting

Please select the one below that is the best fit for your research. If you are not sure, read the appropriate sections before making your selection.

☒ Life sciences ☐ Behavioural & social sciences ☐ Ecological, evolutionary & environmental sciences

For a reference copy of the document with all sections, see nature.com/documents/nr-reporting-summary-flat.pdf

Life sciences study design

All studies must disclose on these points even when the disclosure is negative.

Sample size	No statistical method was used to pre-determine sample size. Sample sizes were indicated in the legend of each Figure and Supplementary Figure.
Data exclusions	No exclusion.
Replication	Western Blotting was performed three times, and similar observation was made for each replicate. One representative result was shown in the Supplementary figures.<br>Sequencing data was confirmed by calculating correlation and reproducibility between replicates.<br>Other experiments, like flow cytometry assay, were replicated at least three times and all attempts at replication are successful.
Randomization	Experimental materials were not divided into random subgroups. Most comparisons were done between WT and mutants.
Blinding	Investigators were not blinded to group allocation during sample collection or analysis, as the information of cell type was essential for the experiment design and analysis.

Reporting for specific materials, systems and methods

We require information from authors about some types of materials, experimental systems and methods used in many studies. Here, indicate whether each material, system or method listed is relevant to your study. If you are not sure if a list item applies to your research, read the appropriate section before selecting a response.

Materials & experimental systems

n/a	Involved in the study	
☐	☒	Antibodies
☐	☒	Eukaryotic cell lines
☒	☐	Palaeontology and archaeology
☒	☐	Animals and other organisms
☒	☐	Human research participants
☒	☐	Clinical data
☒	☐	Dual use research of concern

Methods

n/a	Involved in the study	
☐	☒	ChIP-seq
☐	☒	Flow cytometry
☒	☐	MRI-based neuroimaging

Antibodies

Antibodies used	Anti-CD31-FITC 555445 BD Mouse mAb, IgG FACS 1:100<br>Anti-CD34-APC 555824 BD Mouse mAb, IgG FACS 1:100

March 2021

Anti-CD43-FTIC 315203 BioLegend Mouse mAb, IgG FACS 1:100
Anti-CD43-PE 343204 BioLegend Mouse mAb, IgG FACS 1:100
Anti-CD73-PE 344003 BioLegend Mouse mAb, IgG FACS 1:100
Anti-CD184-APC/Cy7 306527 BioLegend Mouse mAb, IgG FACS 1:100
Isotype Control Antibody-APC 130-113-831 Miltenyi Biotec Mouse mAb, IgG FACS 1:100
sotype Control Antibody-PE 130-113-834 Miltenyi Biotec Mouse mAb, IgG FACS 1:100
Anti-JunB ab128878 Abcam Rabbit mAb, IgG western blot 1:1,000
Anti-Histone H3 A2348 abclonal Rabbit pAb, IgG western blot 1:10,000
Anti-Rabbit IgG 111-035-144 Jackson ImmunoResearch Goat pAb, IgG western blot 1:2000
Anti-JunB 3753S Cell Signaling Technology Rabbit mAb, IgG IP 1:50
Anti-H3K4me3 9727 Cell Signaling Technology Rabbit pAb, IgG ChIP 1:50
Anti-H3K27me3 pAb-069-050 Diagnode Rabbit pAb, IgG ChIP 1:50

Validation

All antibodies used in this study were validated in human cell lines prior to experimentation.

Eukaryotic cell lines

Policy information about cell lines

Cell line source(s)

H1 hESC lines were purchased from WiCell. HUES8 hESC are gifts from Dr. Danwei Huangfu lab.

Authentication

The H1 hESC and HUES8 hESC cell lines were not authenticated.

Mycoplasma contamination

No contamination for all cell lines

Commonly misidentified lines
(See ICLAC register)

No commonly misidentified cell lines were used.

ChIP-seq

Data deposition

☒ Confirm that both raw and final processed data have been deposited in a public database such as GEO.

☒ Confirm that you have deposited or provided access to graph files (e.g. BED files) for the called peaks.

Data access links
May remain private before publication.

ATAC-seq, ChIP-seq and RNA-seq datasets are deposited at the Gene Expression Omnibus repository (accession GSE168372, access token gfwhaqganxwlnkd).

Files in database submission

H3K27me3_EPC_rep1_1.fq.gz
H3K27me3_EPC_rep1_2.fq.gz
H3K27me3_EPC_rep2_1.fq.gz
H3K27me3_EPC_rep2_2.fq.gz
H3K27me3_hPSC_rep1_1.fq.gz
H3K27me3_hPSC_rep1_2.fq.gz
H3K27me3_hPSC_rep2_1.fq.gz
H3K27me3_hPSC_rep2_2.fq.gz
H3K27me3_HPC_rep1_1.fq.gz
H3K27me3_HPC_rep1_2.fq.gz
H3K27me3_HPC_rep2_1.fq.gz
H3K27me3_HPC_rep2_2.fq.gz
H3K27me3_VME_rep1_1.fq.gz
H3K27me3_VME_rep1_2.fq.gz
H3K27me3_VME_rep2_1.fq.gz
H3K27me3_VME_rep2_2.fq.gz
H3K4me3_EPC_rep1_1.fq.gz
H3K4me3_EPC_rep1_2.fq.gz
H3K4me3_EPC_rep2_1.fq.gz
H3K4me3_EPC_rep2_2.fq.gz
H3K4me3_hPSC_1.fq.gz
H3K4me3_hPSC_2.fq.gz
H3K4me3_HPC_1.fq.gz
H3K4me3_HPC_2.fq.gz
H3K4me3_VME_rep1_1.fq.gz
H3K4me3_VME_rep1_2.fq.gz
H3K4me3_VME_rep2_1.fq.gz
H3K4me3_VME_rep2_2.fq.gz
H3K27me3_EPC_peak.bed
H3K27me3_HPC_peak.bed
H3K27me3_hPSC_peak.bed
H3K27me3_VME_peak.bed
H3K4me3_EPC_peak.bed
H3K4me3_HPC_peak.bed

	H3K4me3_hPSC_peak.bed<br>H3K4me3_VME_peak.bed
Genome browser session (e.g. UCSC)	no longer applicable

Methodology

Replicates	2 replicates for H3K27me3 of hPSC, VME, EPC, and HPC. 2 replicates for H3K4me3 of VME and EPC, 1 replicate for H3K4me3 of hPSC and HPC.
Sequencing depth	25~30 milllion of pair-end reads (150bp) per replicate, 60~70% of which (15~20 millions) were uniquely mapped reads.
Antibodies	Anti-H3K4me3 9727 Cell Signaling Technology Rabbit pAb, IgG ChIP 1:50<br>Anti-H3K27me3 pAb-069-050 Diagnode Rabbit pAb, IgG ChIP 1:50
Peak calling parameters	For mapping: bowtie2 with the parameter: --t --q --N 1 --L 25.<br>For peak-calling: macs2 callpeak with the parameter: -p 0.01 -B --SPMR --nomodel --nolambda --shift 73.
Data quality	Data quality was verified by using FastQC (version 0.11.9).<br>The peak number of each sample is as follows (q < 0.05 and fold change >5):<br>H3K27me3-hPSC 9575<br>H3K27me3-VME 18105<br>H3K27me3-EPC 15049<br>H3K27me3-HPC 23284<br>H3K4me3-hPSC 41875<br>H3K4me3-VME 44583<br>H3K4me3-EPC 35205<br>H3K4me3-HPC 29074
Software	Trim_galore (version 0.6.7) was used to remove adaptors.<br>bowtie2 (version 2.3.5) was used to reads mapping.<br>samtools (version 1.9) was used to convert SAM to BAM file.<br>Picard MarkDuplicates (version 2.26.10) was used to remove Duplicate reads.<br>MACS2 (version 2.2.6) was used to call peak of bam without duplicate reads.<br>Homer (version 4.10.4) was used enrich TF motif.<br>deeptools (version 3.3.0)was used to generate signal track.<br>ChromHMM (version 1.22) was used to identify and characterize chromatin states.

Flow Cytometry

Plots

Confirm that:

☒ The axis labels state the marker and fluorochrome used (e.g. CD4-FITC).

☒ The axis scales are clearly visible. Include numbers along axes only for bottom left plot of group (a 'group' is an analysis of identical markers).

☒ All plots are contour plots with outliers or pseudocolor plots.

☒ A numerical value for number of cells or percentage (with statistics) is provided.

Methodology

Sample preparation	Cells were resuspended in a FACS buffer (PBS with 0.1 % BSA and 2.5 mM EDTA), and incubated with antibodies for 30 minutes at 4°C, followed with washed and suspended in 0.1% BSA/PBS buffer.
Instrument	BD FACSAria SORP
Software	FlowJo 10 (version 10.4)
Cell population abundance	The abundance and purity of post-sort populations was described in the result section in the manuscript (see, in particular, Figure 7, Supplementary Figure 6 and Supplementary Figure 7)
Gating strategy	The gating strategy is described in details in supplementary figure 6 and supplementary figure 7. Briefly, all recorded events were gated according to FSC-A and SSC-A. Single cells were selected using FSC-H vs. FSC-W. Further gating on lineage markers (i.e. KDR, CD34, CD31, CD144, CD184, or CD73). Negative controls stained with isotype IgG.

☒ Tick this box to confirm that a figure exemplifying the gating strategy is provided in the Supplementary Information.
